# DR region specific antibody ameliorated but ouabain worsened renal injury in nephrectomized rats through regulating Na,K-ATPase mediated signaling pathways

**DOI:** 10.18632/aging.101815

**Published:** 2019-02-26

**Authors:** Juan Wang, Sayyed Hanif Ullah, Meihe Li, Miao Zhang, Fujun Zhang, Jin Zheng, Xiaofei Yan

**Affiliations:** 1 Department of Biochemistry and Molecular Biology, Medical College of Xi'an Jiaotong University, Xi'an 710061, China; 2 Department of Pathology, Medical College of Xi'an Jiaotong University, Xi'an 710061, China; 3 Department of Pathology, Ankang Central Hostipal, An’kang 725000, China; 4 Hospital of Nephrology, First Affiliated Hospital of Xi’an Jiaotong University, Xi’an 710061, China

**Keywords:** renal damage, DRm217, ouabain, Na^+^-K^+^-ATPase, Src

## Abstract

Reduced Na^+^-K^+^-ATPase function is reported in various renal diseases. This implies that increase of Na^+^-K^+^-ATPase function may be a new target in treatment of renal injury. We previously reported that Na^+^-K^+^-ATPase was stabilized by DRm217, a specific antibody against DR region of Na^+^-K^+^-ATPase. In this study, we compared the protective effect of DRm217 and ouabain on kidney in a chronic kidney disease rat model and investigated the mechanism under it. We found that DRm217 improved renal function, alleviated glomerulus atrophy, inhibited renal tubular cells apoptosis, tubulointerstitial injury and renal fibrosis in 5/6 nephrectomized rats. Contrary to DRm217, ouabain worsened renal damage. Activated Na^+^-K^+^-ATPase /Src signaling pathway, increased oxidant stress and activated inflammasome were responsible for nephrectomized or ouabain-induced renal injury. DRm217 inhibited Na^+^-K^+^-ATPase /Src signaling pathway, retarded oxidant stress, suppressed inflammasome activation, and improved renal function, suggesting a novel approach to prevent renal damage.

## Introduction

Chronic kidney disease (CKD) is a progressive disease which leads to gradual loss of kidney function in all age groups [1]. The precise prevalence of CKD is still not known. But it is estimated that there are approximately 7% young adults and 35% elders suffer from CKD [2]. It leads to progressive renal failure and cardiovascular system diseases, including heart failure [3]. Though much have been learned about the mechanism of CKD, fewer therapeutic strategies have been shown to improve renal outcome. Accordingly, the development of innovative therapies to prevent renal damage in CKD is very important.

The Na^+^-K^+^-ATPase is a ubiquitous transmembrane protein that transports three Na^+^ out of and two K^+^ into the cell for maintaining cell membrane potential and intracellular ion homeostasis [4]. Numerous studies indicate the involvement of Na^+^-K^+^-ATPase in development of many diseases, including renal injury [5,6]. For example, decreased Na^+^-K^+^-ATPase activity has been reported in injured kidney cells, renal damage animal models and even in renal patients [5,7,8]. Furthermore, attenuation of renal injury is usually accompanied by the recovery of Na^+^-K^+^-ATPase function [9,10]. Thus, Na^+^-K^+^-ATPase plays an important role in renal injury.

Cardiotonic steroids such as digoxin, digitalis and ouabain are natural ligands of Na^+^-K^+^-ATPase. They contribute to inhibition of Na^+^-K^+^-ATPase pump function or activation of Na^+^-K^+^-ATPase signal cascade. A recent study shows that there are elevated levels of cardiotonic steroids in patient with chronic renal failure [11]. This information suggests Na^+^-K^+^-ATPase is a potential target for therapy. It is reported that there are decreased Na^+^-K^+^-ATPase activation and decreased Na^+^-K^+^-ATPase expression in the cell membrane, but increased Na^+^-K^+^-ATPase in lysosomes in hyperuricemia-induced renal tubular injury [5]. In previous, our group reports that a specific monoclonal antibodies DRm217 against DR region (Asp 897–Arg 911) of the NKA a-subunit stimulate NKA activity, stabilize the membrane expression of Na^+^-K^+^-ATPase and inhibit Na^+^-K^+^-ATPase endocytosis [12,13]. In the present study, we try to compare the effect of DRm217 and ouabain on kidney in a CKD rat model and investigate the mechanisms under it.

## RESULTS

### DRm217 improved and ouabain worsened renal function

**Figure 1:**
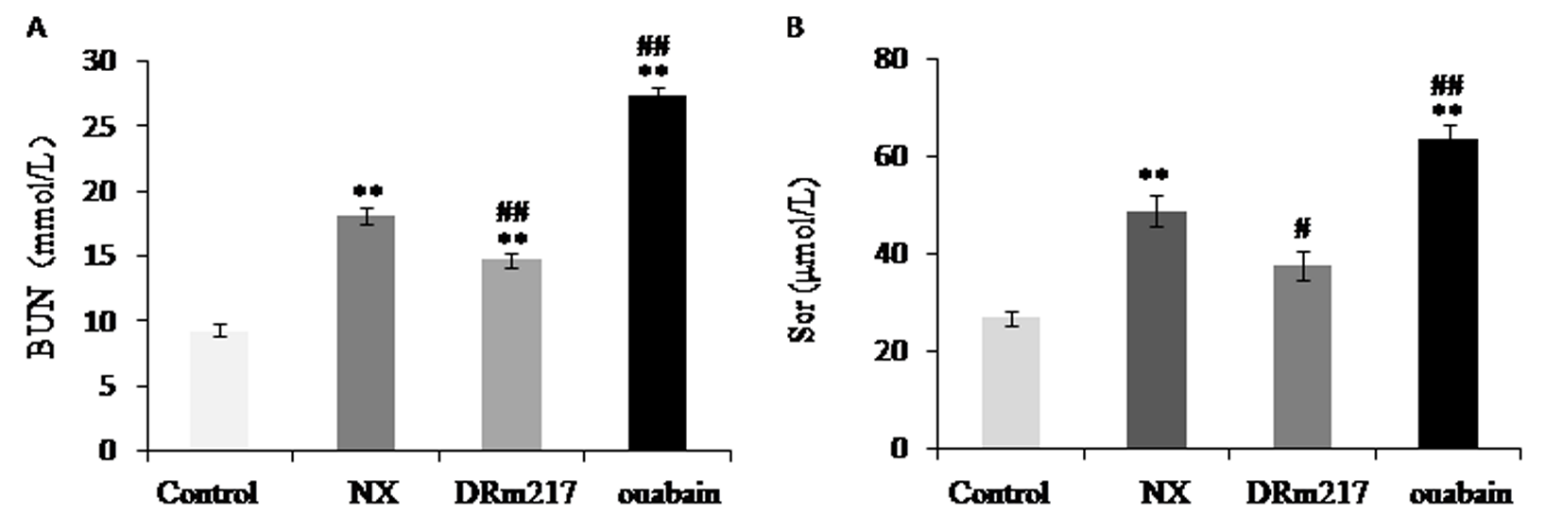
**DRm217 improved and ouabain worsened renal function.** Serum BUN content (**A**) and serum creatinine content (**B**) were detected. The increased serum BUN and creatinine levels were blunted by DRm217 treatment. In contrast, ouabain treatment elevated these parameters. Means±SEM, n=6-8; ** *p*<0.01, vs control group; # *p*<0.05, ## *p*<0.01, vs NX group.

As expected, after 4 weeks of 5/6 renal ablation, rats developed renal failure, characterized by increased serum BUN (Figure 1A) and serum creatinine (Figure 1B). The increased serum BUN and creatinine levels were blunted by DRm217. In contrast, ouabain elevated these parameters.

### DRm217 alleviated but ouabain strengthened kidney morphological damage

**Figure 2:**
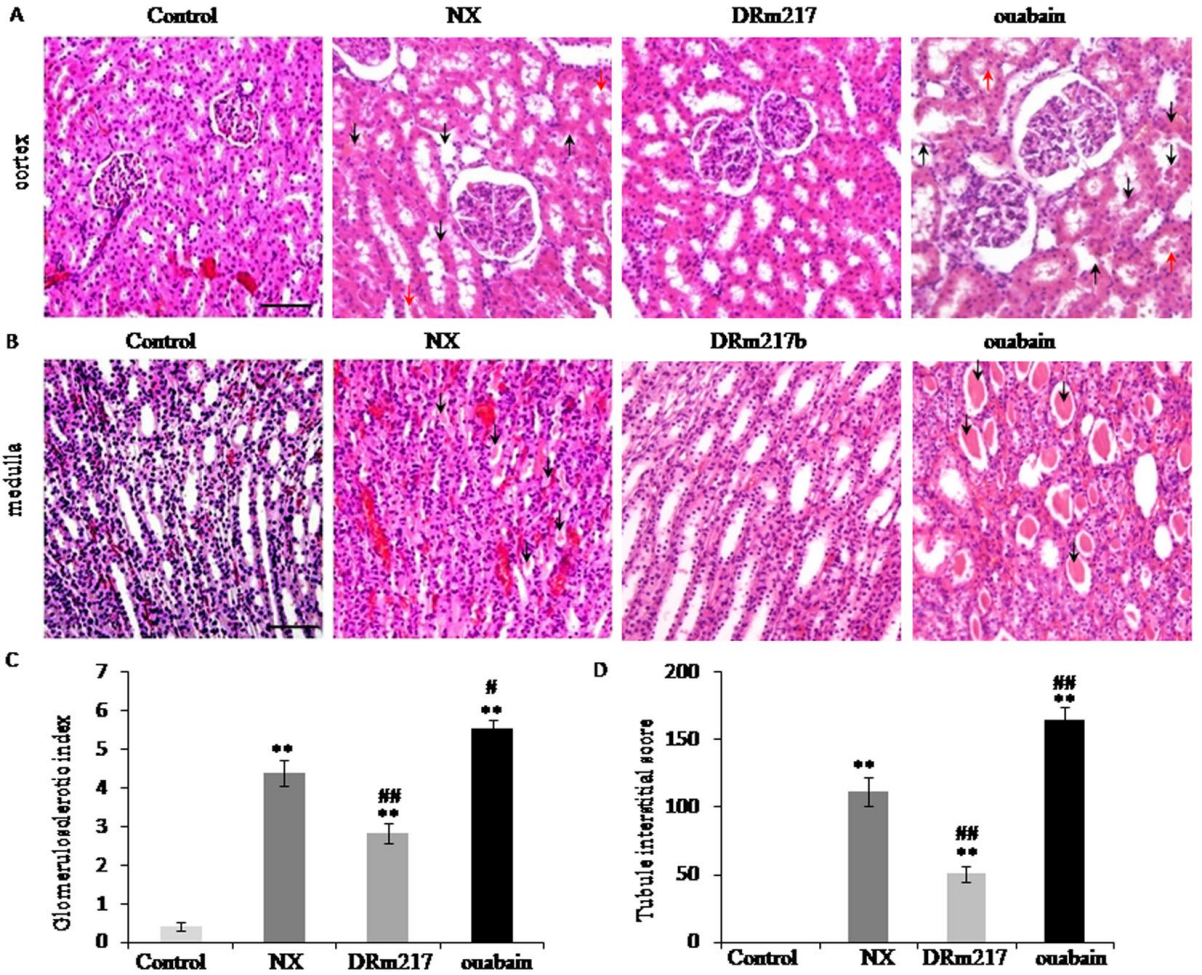
**DRm217 alleviated but ouabain strengthened kidney morphological damage.** (**A**) Represent images of HE staining for renal cortex. Representative photomicrographs of glomerulosclerosis, tubular dilation and degeneration (black arrow), and protein deposition (red arrow) were observed. (**B**) Represent images of HE staining for renal medulla. Representative photomicrographs of muddy brown casts (black arrow) were observed. (**C**) Calculated results of glomerulosclerotic index. (**D**) Calculated results of Tubule interstitial score. n=6-8. Means±SEM; *** *p*<0.001, vs control group; # *p*<0.05, ## *p*<0.01, ###*p*<0.001, vs NX group. Scale bar, 100 μm.

In comparison with sham control, nephrectomized rats demonstrated pathologic features of renal injury. The renal injury included glomerular sclerosis, glomerular compensatory hypertrophy, nephric tubular degeneration, and protein deposition in renal tubular lumen. In contrast, DRm217 reduced but ouabain promoted the histologic features of renal injury (Figure 2A). We also found the muddy brown casts in the inner medulla region in the slides of NX group and ouabain group. There were no casts were found in DRm217 group (Figure 2B). Histological score further confirmed the observation. Compared with sham-operated animals, nephrectomized rats have high Glomerulosclerotic index and Tubule interstitial score. However, DRm217 significantly reduced but ouabain promoted these histological score (Figure 2C, D). This information further confirmed that DRm217 alleviated but ouabain strengthened renal injury.

### DRm217 attenuated but ouabain strengthened renal fibrosis

**Figure 3:**
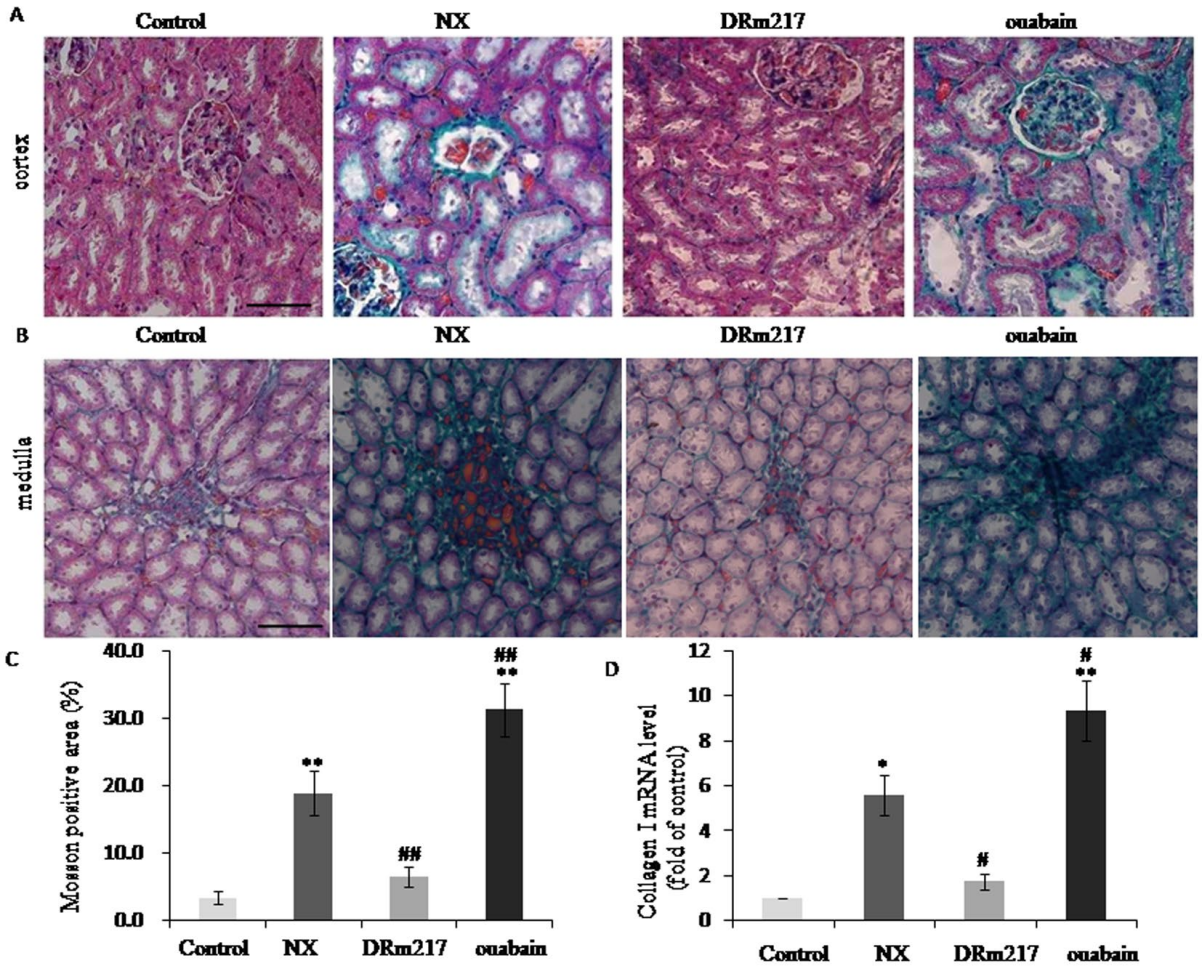
**DRm217 attenuated but ouabain strengthened renal fibrosis.** (**A**) Represent images of Masson staining for renal cortex. (**B**) Represent images of Masson staining for renal medulla. (**C**) Quantitative analysis of Masson-staining area. (**D**) RT-qPCR analysis of mRNA levels of Collagen I. n=6-8. Means±SEM; * *p<0.05*, **p<0.01, vs control group (Con); # *p*<0.05, ## *p*<0.01, vs NX group. Scale bar, 100 μm.

Renal fibrosis area was measured by Masson staining. As expected, there were much Masson positive staining in either renal cortex or inner medulla region in the rats of NX group or ouabain group. In contrast, rats of DRm217 group demonstrated reduced Masson positive staining area (Figure 3A, B). The percentage of fibrosis area was 18.94 ± 3.31% in NX groups and 31.4 ± 3.96% in ouabain group. DRm217 significantly reduced fibrosis area to 6.51 ± 1.46% (Fig. 3C). Collagen I is one of common used fibrosis markers. The mRNA levels of ColIa were increased in NX rats. DRm217 reduced but ouabain increased ColIa mRNA level (Fig 3. D). These data indicated that DRm217 attenuated but ouabain strengthened renal fibrosis.

### DRm217 attenuated but ouabain strengthened renal tubular cells apoptosis

**Figure 4:**
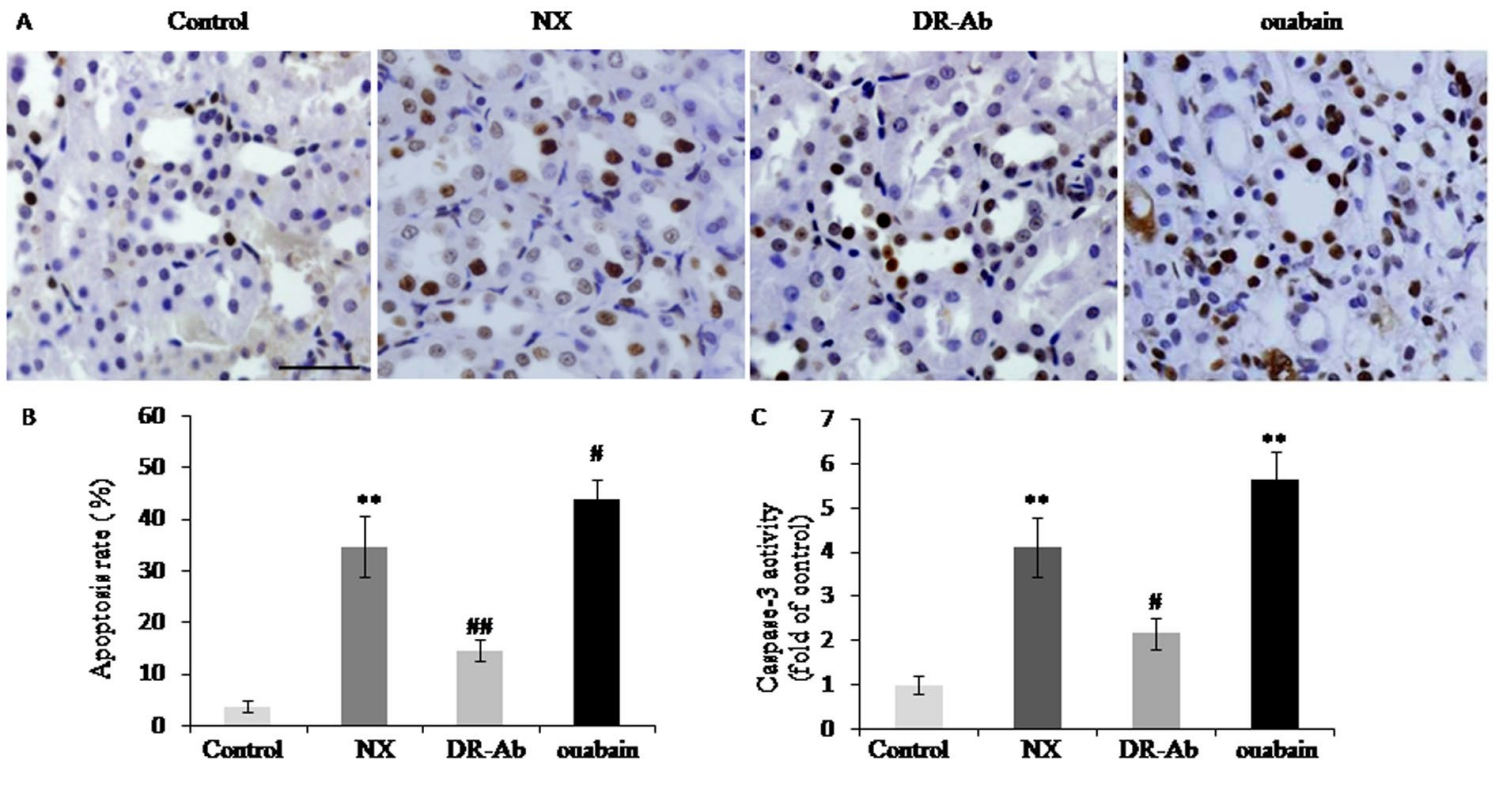
**DRm217 attenuated but ouabain strengthened renal tubular cells apoptosis.** (**A**) Represent images for TUNEL staining. (**B**) Statistical result of TUNEL positive cells. (**C**) Caspase 3 activity in different-treated renal tissues. n=6-8. Means±SEM; * *p<0.05*, **p<0.01, vs control group; # *p*<0.05, ## *p*<0.01, vs NX group. Scale bar, 100 μm.

TUNEL assay was used for assessing cell apoptosis. Compared with control, the TUNEL positive cells was 34.64 ± 5.85% in NX group and 43.68± 4.06% in ouabain group. In contrast, DRm217 significantly decreased apoptosis rate to 14.60 ± 2.17% (Fig4. A, B). Further analysis also found that caspase-3 activity was increased in the renal tissues in NX group and ouabain group. DRm217 retarded the increase of caspase-3 activity. These results confirmed that DRm217 attenuated but ouabain strengthened renal tubular cells apoptosis (Fig. 4C).

### DRm217 blocked Src activation in kidney of 5/6 nephrectomized rats

**Figure 5:**
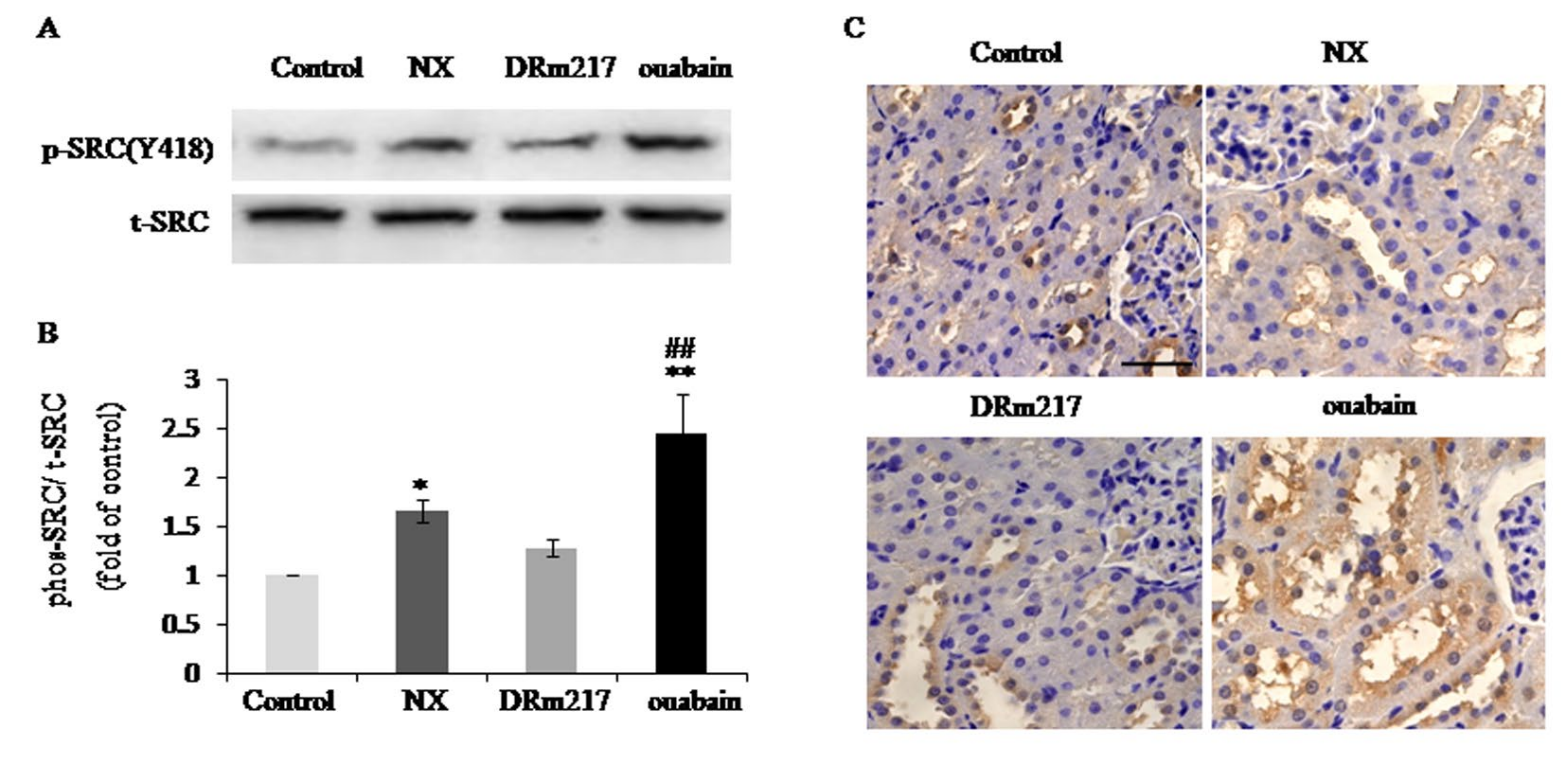
**DRm217 blocks Src activation in kidney of 5/6 nephrectomized rats.** (**A**) Representative western blots for Src(Y418) phosphorylation. (**B**) Quantitative analysis for Src(Y418) phosphorylation. (**C**) Immunohistochemical analysis of phosphorylated Src in renal tissues. n=6-8. Means±SEM; * *p<0.05*, **p<0.01, vs control group (Con); # *p*<0.05, ## *p*<0.01, vs NX group. Scale bar, 100 μm.

Previous studies showed that Src is activated by Na^+^-K^+^-ATPase [14] and activation of Src plays an important role in renal injury [15]. In the present study, we found phosphorylated Src, the active form of Src, was elevated in the kidneys of NX rats (figure 5A). DRm217 significantly reduced Src activation (figure 5A). On the contrary, ouabain increased Src activation (figure 5A). Immunohistochemistry results further revealed that phosphorylated Src was elevated in renal tissues of NX group and ouabain group. Phosphorylated Src mostly focus on the apical membrane of renal tubular cells (figure 5B).

### DRm217 attenuated but ouabain strengthened oxidative stress

**Figure 6:**
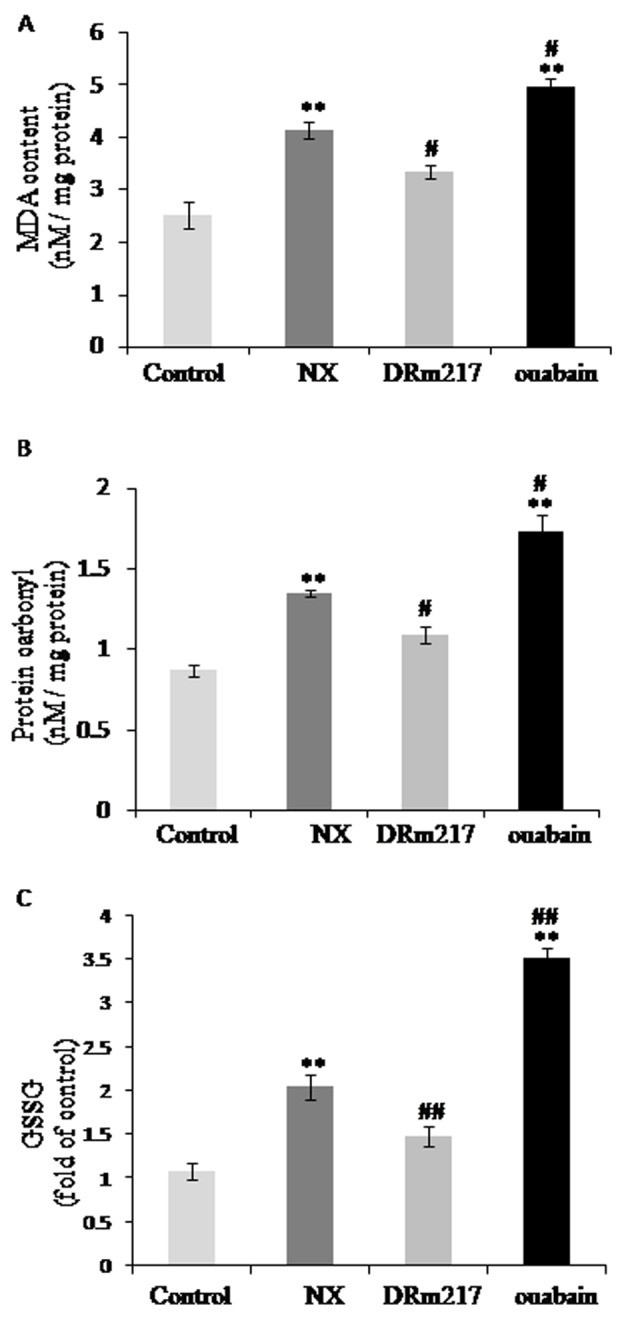
**DRm217 attenuated but ouabain strengthened oxidative stress.** Contents of Malondialdehyde contents (**A**), protein carbonyl contents (**B**), and oxidized glutathione (**C**) in different renal tissues. DRm217 treatment significantly attenuated but ouabian enhanced the content of malondialdehyde, protein carbonyl and oxidized glutathione in the renal tissues of 5/6 nephrectomized rats. n=6-8. Means±SEM; * *p<0.05*, **p<0.01, vs control group (Con); # *p*<0.05, ## *p*<0.01, vs NX group.

Oxidative stress was reflected by the levels of malondialdehyde, protein carbonylation and oxidized glutathione. The content of these factors were increased in the kidney of NX rats (Fig.6 A-C). DRm217 significantly attenuated but ouabian enhanced the content of malondialdehyde, protein carbonyl and oxidized glutathione (Fig.6 A-C). These data suggested that there were increased oxidative stress in 5/6 nephrectomy. Ouabain increased and DRm217 decreased oxidative stress.

### DRm217 attenuated but ouabain strengthened NLRP3 inflammasome activation

**Figure 7:**
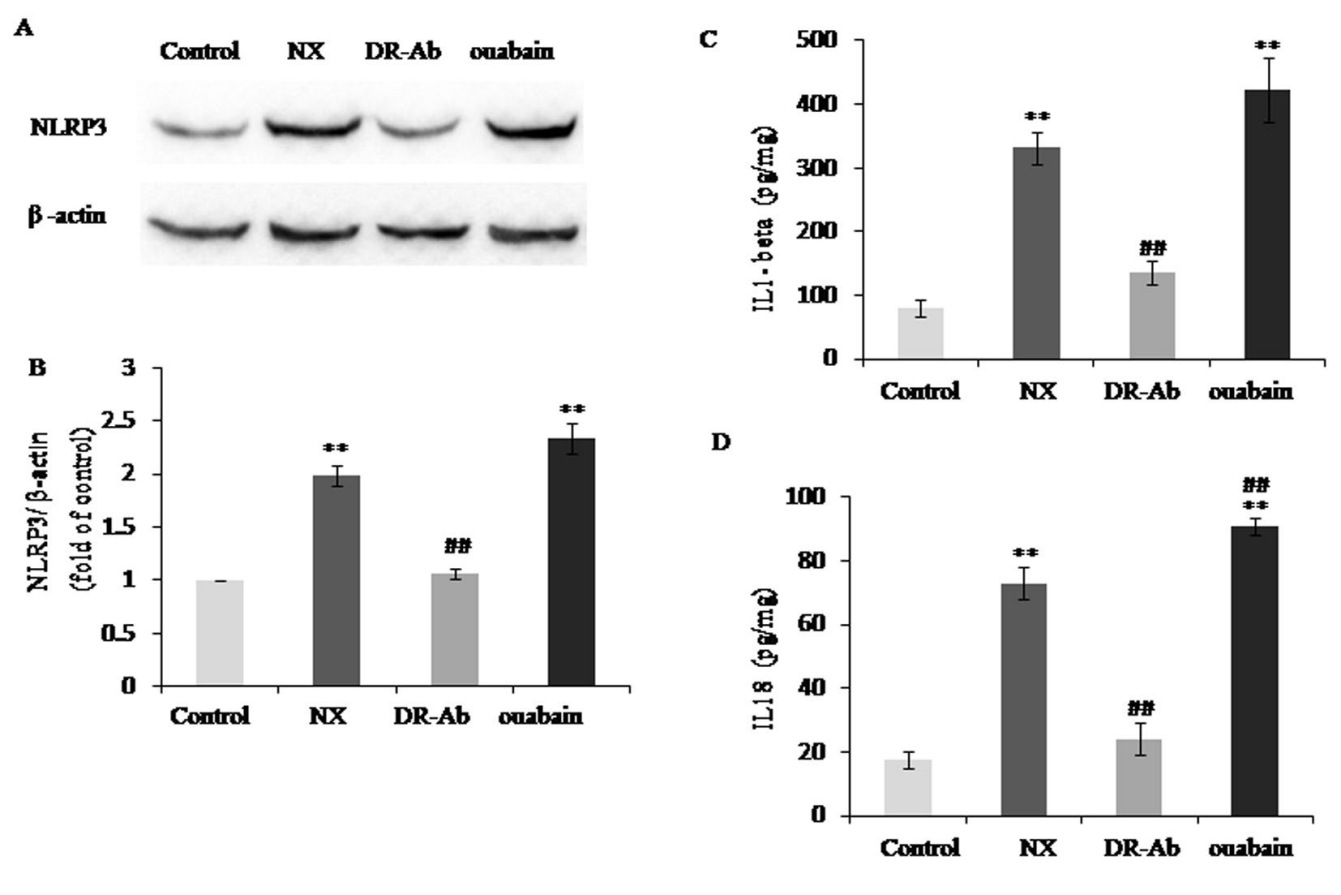
**DRm217 attenuated but ouabain strengthened inflammation in renal tissues of 5/6 nephrectomized rats.** (**A**, **B**) Representative western blotting image and quantitative analysis of NLRP3. (**C, D**) ELISA analysis of protein levels of IL-1β and IL-18. n=6-8. Means±SEM; * *p<0.05*, **p<0.01, vs control group (Con); # *p*<0.05, ## *p*<0.01, vs NX group.

NLRP3 inflammasome activation is closely related to chronic kidney disease [16]. IL-1β and IL-18 are two inflammasome-related cytokines that released by activated NLRP3 inflammasome [17]. The protein levels of NLRP3 protein (figure 7A), IL-1 β (figure 7A) and IL-18 (figure 7C) were significantly increased in the kidneys of NX rats. DRm217 significantly reduced NLRP3 protein, IL-1 β and IL-18 expression (figure 7A-C). On the contrary, ouabain increased NLRP3 protein, IL-1 β and IL-18 expression (figure 7A-C).

### Inhibition of Src attenuated AngII effect on increasing of collagen I, malondialdehyde and IL-1β in HK-2 cell

**Figure 8:**
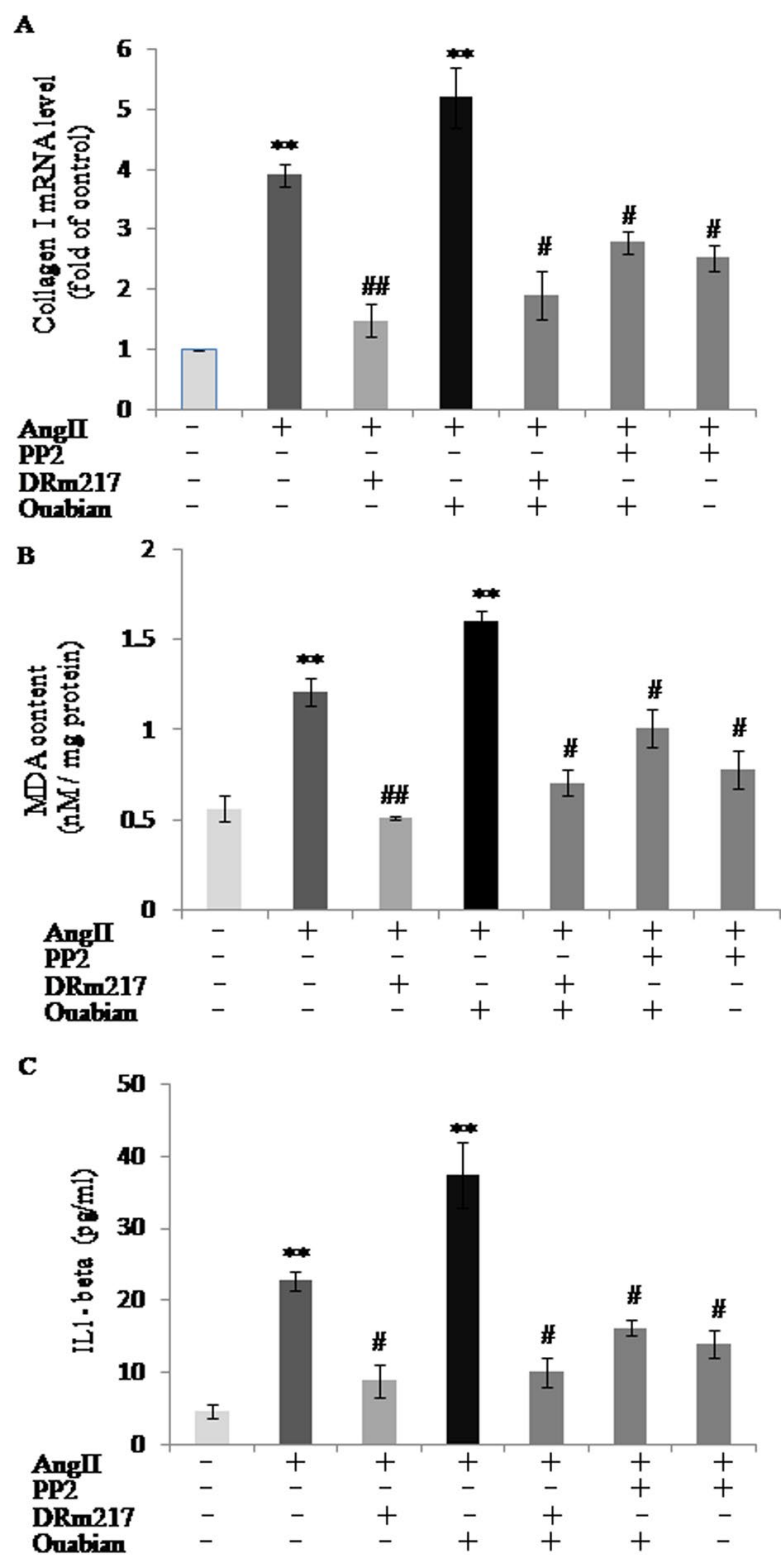
**DRm217 and PP2 attenuated but ouabain strengthened AngII effect on increasing of collagen I, malondialdehyde and IL-1β in HK-2 cell.** mRNA level of collagen I (**A**), contents of malondialdehyde contents (**B**), and contents of IL-1β (**C**) in different-treated cells. AngII increased the expression of collagen I, malondialdehyde and IL-1β, whereas, DRm217 and PP2 decreased but ouabian increased the expression of collagen I, malondialdehyde and IL-1β in AngII-treated cells. n=4. Means±SEM; * *p<0.05*, **p<0.01, vs control group (Con); # *p*<0.05, ## *p*<0.01, vs AngII group.

To mimic the overactivation of intrarenal RAS in the remnant kidney, AngII was added to HK-2 cell culture medium for *in vitro* study. AngII increased the expression of collagen I, malondialdehyde and IL-1β, whereas, DRm217 decreased but ouabian increased the expression of collagen I (figure 8A), malondialdehyde (figure 8B) and IL-1β (figure 8C) in AngII-treated cells. PP2, a specific inhibitor of Src, also decreased AngII and ouabain -induced collagen I, malondialdehyde and IL-1β overexpression (figure 8A-C).

## DISCUSSION

CKD is characterized by an irreversible deterioration of renal function, with glomerular atrophy, glomerular compensatory hypertrophy, nephric tubular degeneration, and renal fibrosis as the final common pathologic change. Identify treatment targets for slowing CKD development is a hot topic in renal research. In the present study, we demonstrated that DRm217, a specific antibody against DR region of Na^+^-K^+^-ATPase, restored renal function in 5/6 nephrectomized rats. We showed that DRm217 administration also attenuated glomerulus atrophy, renal tubular cells apoptosis, tubulointerstitial injury and renal fibrosis in the injured kidney. Interestingly, ouabain, a natural and classical ligand of Na^+^-K^+^-ATPase, deteriorated renal injury in 5/6 nephrectomized rats. Collectively, these data suggested that Na^+^-K^+^-ATPase acts as a key mediator in the process of renal disease.

Addition to it is classical ion pump function, Na^+^-K^+^-ATPase also functions as a scaffold and signaling transducer [18]. Src is an important kinases associating with Na^+^-K^+^-ATPase [19,20]. Activated Src plays important roles in renal injury and fibrosis [14,21,22]. Our results clearly showed that DRm217 significantly ameliorated Src activation in 5/6 nephrectomized rats. However, ouabain stimulated Src activation and increased renal injury. It has been proved that endogenous ouabain (or ouabain-like factors) is elevated in patients with terminal renal failure [23,24] and in renal injury –related rat models [25,26]. The binding of endogenous ouabain to the α-subunit of the Na^+^-K^+^-ATPase may directly initiate Na,K-ATPase signaling cascades and contribute to renal failure [27]. It is interesting to note that pNaKtide, a cell permeate analog of Na/K-ATPase, also ameliorated renal interstitial fibrosis [28]. Though both DRm217 and pNaKtide could inhibit Src activation, the mechanism under it is difference. Our previous studies showed that DRm217 inhibit Src activation via stabilizes Na^+^-K^+^-ATPase membrane [13], but pNaKtide inhibit Src activation via binds to the kinase domain of Src [29]. We also showed that phosphorylated Src mostly focus on the apical membrane of renal tubular cells. Whether this specific location has some function on renal injury needs further investigation.

Oxidant stress plays a dominant role in the pathophysiological event of chronic renal disease [30,31]. Hemodialysis and CKD patients exhibit impaired mitochondrial respiration and increased oxidative stress [32]. Inflammation also plays a detrimental role in the development of renal fibrosis. Persistent low-grade inflammation supposedly acts as a pre-conditioning event in the development of renal fibrosis in diabetic nephropathy [33]. It was found that IL-1β and IL-18, two inflammatory cytokines, was up-regulated in subtotal nephrectomy or hyperuricemia-induced renal tubular injury [34,35]. Of note, Na^+^-K^+^-ATPase has functions on mediating inflammation and oxidative stress through Na^+^-K^+^-ATPase /Src signaling cascade [5,36]. So we moved on to examine whether oxidant stress and inflammation have roles in different renal effects mediated by DRm217 and ouabain. Levels of malondialdehyde, protein carbonyl and oxidized glutathione were significantly reduced by DRm217, but strengthened by ouabain, suggesting DRm217 alleviates but ouabain aggravated oxidative stress in 5/6 nephrectomized rats. Increased expression of NPRL-3, IL-18 and IL-1β in nephrectomized rats was also significantly reduced by DRm217, indicating that DRm217 exerted protective effects partly through anti- inflammasome activation. As expected, ouabain increased NLPR-3, IL-18 and IL-1β expression.

Src is a splicer in Na^+^-K^+^-ATPase signaling. In vitro cell model proved that inhibiting Src by PP2 partly inhibited the production of collagen, malondialdehyde and IL-1β in AngII- induced renal cell. These results implied that Src activation have functions on renal fibrosis, oxidative stress and inflammation. As our *in vivo* model proved that DRm217 significantly ameliorated Src activation, we concluded DRm217 also exerted its protective function partly through inhibiting Src activation. However, the inhibition effect on the expression of collagen, malondialdehyde and IL-1β are not coincided between PP2 and DRm217 treatment. This phenomenon implies that there has other mechanism except inhibition of Src activation under DRm217’s protective function.

In summary, this study demonstrated that DRm217 improved renal function, attenuated glomerulus atrophy, renal tubular cells apoptosis, tubulointerstitial injury, renal fibrosis in 5/6 nephrectomized rats. Whereas, ouabain made renal damage worsen. Na^+^-K^+^-ATPase /Src signaling pathway, oxidant stress and inflammasome activation contributed to nephrectomized and ouabain-induced renal injury. DRm217 exerted its protective effect via inhibiting Na^+^-K^+^-ATPase /Src signaling pathway and retarding oxidant stress and inflammasome activation. Targeting Na^+^-K^+^-ATPase could be a novel approach for the treatment of chronic renal failure.

## MATERIALS AND METHODS

### Chemicals and reagents.

All chemicals, including ouabain were purchased from Sigma–Aldrich (St. Louis, MO). Primary antibodies to Src (Tyr(P)418) was purchased from Invitrogen (California, USA). Primary antibodies to β-actin, total-Src and NLRP3 were purchased from ProteinTech Company (Chicago, USA). HRP-labeled goat anti-mouse, goat anti-rabbit antibody, and Bicinchoninic acid (BCA) assay kit were purchased from Pierce Company (Pierce Biotechnology, Rockford, IL). Normal mouse IgG was purchased from Bioss Biotechnology Company (Beijing, China). DRm217 monoclonal antibody was purified from mice ascites by HiTrap Protein G HP columns (GE Company) in our lab.

### Animals protocols

(1) Male Sprague Dawley rats, 7-week-old, weighing 225–250 grams, were used in this study. All animal care and experimental procedures were approved by Xi'an Jiaotong University Committee on Animal Care. All the experiments conformed to the international guidelines on the ethical use of animals. (2) For subtotal (5/6) nephrectomy, rats were anesthetized by 3% sodium pentobarbital (30 mg/kg body weight, i.p). The right kidney and two thirds of the left kidney were surgically removed as previously described [37]. This model has been widely used as a classic model of chronic renal disease [37]. The animals were separated into four groups: ①Sham control group (n = 5), rats were subjected to anesthesia and manipulation of the renal pedicles; ②NX group (n = 6): rats were subjected to 5/6 nephrectomy and treated with normal mouse IgG (2mg/Kg/every other day, intraperitoneal); ③DRm217 group (n = 8): rats were subjected to 5/6 nephrectomy and treated with DRm217 (2mg/Kg/every other day, intraperitoneal); ④Ouabain group (n = 8): rats were subjected to 5/6 nephrectomy and treated with ouabain (30ug/Kg/every other day, intraperitoneal). All the treatment were done from the second day after 5/6 nephrectomy. All animals were sacrificed 4 weeks after the onset of treatments. Serum and kidney were collected.

### Detection of serum creatinine and blood urea nitrogen

Blood was extracted via the abdominal aorta and serum was obtained by centrifugation at 4000 rpm for 10 min. Serum creatinine (Scr) and blood urea nitrogen (BUN) were determined using a Hitachi 7060 chemistry analyzer.

### Hematoxylin and eosin staining

Kidney tissue was fixed in 10% formalin, embedded in paraffin. Tissue sections (5 𝜇m thick) were cut and stained with hematoxylin-eosin for histopathological evaluation. Samples were analyzed by a pathologist blinded to the experimental group to which the rat belonged. Glomerulosclerotic Index (GSI) was evaluated as Maric C described [38]. Briefly, one hundred glomeruli per section were randomly selected and the degree of glomerular damage assessed using a semiquantitative scoring method: grade 0, normal glomeruli; grade 1, sclerotic area up to 25% (minimal sclerosis); grade 2, sclerotic area 25 to 50% (moderate sclerosis); grade 3, sclerotic area 50 to 75% (moderate-severe sclerosis); grade 4, sclerotic area 75 to 100% (severe sclerosis). The glomerulosclerotic index (GSI) was calculated using the following formula: GSI = (1 × n1) +(2 × n2) +(3 × n3) + (4×n4)/100, where nx is the number of glomeruli in each grade of glomerulosclerosis. 100 sections were examined for each kidney. The GSI for each kidney is an average value of all 100 sections. Tubule interstitial score (TIS) was evaluated by determining the percentage of tubules in the cortex and medulla in which epithelial necrosis or necrotic debris was observed. Briefly, 100 sections were examined for each kidney and a score from 0 to 3 was given for each tubular profile: 0, normal histology; 1, with up to one-third of tubular profile showing tubular cell swelling and brush border loss; 2, as for score 1 but greater than one-third and less than two-thirds of tubular profile showing tubular cell swelling and brush border loss; and 3, greater than two-thirds of tubular profile showing tubular cell swelling and brush border loss. The total score for each kidney was calculated by addition of all 100 scores with a maximum score of 300.

### Masson staining

Masson staining was used for analyzing renal fibrosis. Briefly, paraffin-embedded renal sections were deparaffinized and rehydrated. Then the sections were stained with 0.1% Masson staining buffer to evaluate the collagen deposition. The respective Masson stained area (green, fibrosis) and non-Masson stained (red, normal) areas of the sections were measured digitally using Image-J software (Media Cybernetic, USA) based on research of Shu J et al [39]. The fibrotic area= non-Masson stained area /total area×100%.

### TUNEL assay

In Situ Cell Death Detection Kit was purchased from Zhongshan Jinqiao Biotechnology (Cat NO: ZK-8005, Beijing, China). TUNEL assay was conducted according to the manufacturer’s instructions. Briefly, deparaffinization and rehydration were done before paraffin-embedded sections were incubated with 3% hydrogen peroxide for 10 min. The slides were then treated with Proteinase K working solution at 37°C for 15min. Then, the sections were washed with PBS twice for 3 min each. Sections were then incubated with TUNEL reaction mixture for 60 min and subsequently with Converter-POD solution for 30 min. The slides were visualized using diaminobenzidine and counterstained with hematoxylin before microscopy. In this method, the apoptotic nuclei were stained dark brown. Normal nuclei were stained blue. Samples were analyzed by a pathologist blinded to the experimental group to which the rat belonged. The results were scored semi-quantitatively by averaging the number of apoptotic cells/field at 400×magnification. Five fields were evaluated per tissue sample. The apoptosis index (AI) = the number of TUNEL-positive cells/the total number of cells counted ×100%.

### Caspase-3 activity assay

Caspase 3 activity was measured by using Caspase 3 Activity Kit (Cat No: C1115, Beyotime Biotechnology Company, Shanghai, China) according to the manufacturer’s instructions. Briefly, renal tissues were put in lysis buffer, homogenized by glass homogenizer and left on ice for 5 min. The lysate was centrifuged at 16,000 g at 4 °C for 15 min. Activities of caspase-3 was measured using substrate peptides Ac-DEVD-pNA. The release of p-nitroanilide (pNA) was qualified by determining the absorbance with a spectrometer plate reader at 405 nm. The increase in activity was calculated as the ratio between values obtained from treated samples versus those obtained in normal controls.

### Western blot

Protein samples were separated by SDS-PAGE and transferred on to a nitrocellulose membrane (Thermo Scientific). After blocking with 10% milk/TBST buffer, the membrane was incubated with various primary antibodies at 4°C overnight. Membranes were then washed three times in TBST buffer, followed by incubation with 1:10,000 dilutions of horseradish peroxidase-conjugated anti-rabbit IgG at room temperature for 1 h and washed three times in TBST. Visualization was carried out using an enhanced chemiluminescence kit (GE Healthcare). The blot after ECL was captured by Chemiluminescence imager (G: BOX Chemi XRQ, Syngene). The density of the bands was quantified by densitometry analysis of the imaged blots using Image J software.

### Immunohistochemistry

Paraffin-embedded sections undergone deparaffinization and rehydration were immersed in citric buffer (0.01 M, pH 10.0) and then incubated with 3% H_2_O_2_ for 10 min. Then the slides were soaked with rabbit serum for 30 min at room temperature. Various primary antibodies (1: 100) was mounted on the slides and incubated at 4°C overnight. After that, HRP conjugated goat anti-rabbit antibody was mounted on the slides. After washed, the samples were stained with DAB and counterstained with hematoxylin.

### Malondialdehyde (MDA) content, Protein carbonyl content and Oxidized glutathione determination

Contents of malondialdehyde, protein carbonyl and oxidized glutathione were determined by Malondialdehyde Assay Kit (Cayman Chemical Co, Ann Arbor, USA), Protein Carbonyl Colorimetric Assay Kit (Cayman Chemical Co, Ann Arbor, USA), and Oxidized glutathione assay kit (Nanjing Jiancheng Bioengineering Institute) according to the manufacturer’s instructions.

### Detection of inflammatory factors by ELISA assay

Renal tissues were treated with homogenizer. The supernatant was separated after centrifugation at 20000g for 15 min at 4 °C. For detected inflammation factors in cell culture medium, cell culture medium was collected after centrifugation at 1000g for 10 min at RT. Levels of IL-1β (BGK5BKB0 Peprotech Company, Rocky Hill, USA for renal tissues; CSB-E08053h CUSABIO company, Wuhan, China for cell culture medium) and IL-18 (Cat No: CSB-E046 10r, CUSABIO company, Wuhan, China) were tested using ELISA test kits. The operations were carried out according the kit instructions. Briefly, samples were placed into wells of the plate and the plate was then incubated for 30 min at 37 °C. The liquids in the wells were removed, and the plate was washed by detergents for 5 times. Then enzyme labeling reagents (50 μL) were added to the wells and incubated for 30 min at 37 °C. After that, the liquids in the wells were removed, and the plate was re-washed by detergents for 5 times. Then Chromogenic agent A (50 μL) and Chromogenic agent B (50 μL) were added to each well one by one. The plate was incubated at 37 °C for 15 min and 50 μL stop buffer was added into each well. The absorbance at 450 nm was measured using a spectrophotometric plate reader (Safire2, Tecan Group Ltd, Switzerland). The concentration standard curve was plotted and the sample concentration was recorded according to the standard curve.

### Cell culture and treatment

The Human tubular epithelial HK2 cell line came from Cell Center of Shanghai Institutes for Biological Sciences. Cells were grown in Dulbecco’s modified Eagle’s medium (DMEM) supplemented with 1% penicillin/streptomycin and 10% fetal bovine serum (FBS) and incubated at 37^o^C in 5% CO_2_. This basic medium was replenished every 3 days. For cell treatment, cells were seeded into 60mm dishes and incubated until they reached about 70% confluence. The cultures were exposed to AngII (Sigma) in a final concentration of 100 nM for 24h. DRm217 (1μM) and ouabain (100 nM) were added 15min after AngII addition. PP2(15 μM) was added 15min before AngII addition.

### RT-qPCR

Total RNA from cells or kidney was isolated separately using RNeasy Mini Kit (Qiagen, Valencia, USA). One microgram of total RNA was reverse transcribed separately into cDNA using the iScript cDNA Synthesis Kit (Bio-Rad, Hercules, USA). After reverse transcription, RT-qPCR was performed on a QuantStudio® 3 (Applied Biosystems, Massachusetts, USA) using SYBR Green quantitative PCR master mix (Takara, Dalian, China) according to the manufacturer instructions. All amplifications were normalized to β-actin. Data were analyzed using the comparison Ct (2−∆∆Ct) method and expressed as fold change compared to the corresponding control. The primer sequences used for the RT-qPCR assay were shown as follows: β-actin forward: GAGGGAAATCGTGCGTGAC; β-actin reverse: GCATCGGAACCGCTCATT; Collagen I forward (rat): CACCCTCAAGAGCCTGAGTC; Collagen I reverse (rat): GTTCGGGCTGATGTACCAGT; Collagen I forward (human): AGTGGTTTG GATGGTGCCAA; Collagen I reverse (human): GCACCATCATTTCCACGAGC.

### Statistical analysis

The data were expressed as the mean ± the standard error of the mean. Comparison of multiple groups was performed by one-way analysis of variance followed by Tukey’s post hoc test. A probability level of less than 0.05 was used to establish significance.
